# A Home‐Based Online Psychoeducation Programme on Subjective Well‐Being Amongst Community‐Dwelling Older Adults With Frailty: A Pilot Randomised Controlled Trial

**DOI:** 10.1111/opn.70079

**Published:** 2026-04-16

**Authors:** An Tao, Helen Yue Lai Chan, Sarah Xiao, Ken Hok Man Ho

**Affiliations:** ^1^ The Nethersole School of Nursing, Faculty of Medicine The Chinese University of Hong Kong Shatin Hong Kong; ^2^ School of Nursing& Midwifery La Trobe University Melbourne Victoria Australia

**Keywords:** frailty, mental health, psychoeducation, subjective well‐being, telehealth

## Abstract

**Aim:**

To evaluate the feasibility and acceptability of a home‐based online psychoeducation (HOPE) programme amongst community‐dwelling older adults with frailty.

**Design:**

This study adopted a single‐blinded, two‐arm randomised controlled trial design.

**Methods:**

Participants were randomly assigned to either the intervention or control group. All participants received 60‐min weekly group‐based online sessions at home via a videoconferencing platform for 12 weeks. The intervention group received psychoeducation sessions guided by self‐determination theory to empower the participants to cope with frailty proactively. By contrast, the control group received educational sessions for general health management. Feasibility was assessed based on eligibility, recruitment and attrition rates; acceptability was evaluated through attendance rate, satisfaction survey and qualitative interviews. Physical and psychosocial outcomes were measured at baseline and 12 weeks post‐allocation. The Mann–Whitney U test and Wilcoxon signed‐rank test were performed for data analysis, as appropriate. Interview transcripts were analysed using qualitative content analysis.

**Results:**

Twenty‐four participants were recruited with a mean age of 77.7 years (SD = 6.2), and most of them (91.7%) were female. The study demonstrated good feasibility with eligibility, recruitment and attrition rates of 55.8%, 82.8% and 16.7%, respectively. The mean satisfaction score was 4.6 out of 5. Participants experienced physical and psychological benefits, despite some practical challenges. Improvements in subjective well‐being and agility were observed in the intervention group across 12 weeks, and the intervention group showed significantly higher subjective well‐being compared to the control group at 12 weeks.

**Conclusion:**

The HOPE programme is feasible and acceptable for community‐dwelling older adults with frailty. Suggested improvements include technical support during sessions, flexible scheduling, practical examples and content consolidation.

**Implications for Practice:**

Home‐based online delivery mode is a feasible, acceptable for providing psychological support for older adults with frailty. To optimise participation, practitioners should provide technical training and flexible scheduling that accounts for individual health fluctuations.

**Reporting Method:**

This study adhered to the CONSORT guidelines for pilot and feasibility trials.

**Patient or Public Contribution:**

No patient or public contribution.

**Trial Registration:**

WHO Primary Registry–Clinical Trials Registry Identified: NCT06415617

## Abstract (highlights)

**Summary**

**What does this research add to existing knowledge in gerontology?**

- This study is the first to demonstrate that a home‐based online psychoeducation programme is feasible and acceptable for community‐dwelling older adults with frailty.
- Guided by self‐determination theory, the programme shows potential to improve subjective well‐being amongst older adults with frailty, extending evidence for theory‐informed psychosocial approaches in this population.
- This study identifies implementation barriers specific to the home‐based online psychoeducation programme, including low digital literacy, schedule conflicts and difficulties with content comprehension and retention.

**What are the implications of this new knowledge for nursing care for and with older adults?**

- Nurses could use online delivery methods to improve access to psychological support for older adults with frailty.
- Flexible schedules and consideration of health fluctuation could facilitate participation in remote group‐based nursing interventions for older adults with frailty.
- Psychoeducation for older adults with frailty should include practical instructions and ongoing review to support comprehension and engagement.

**How could the findings be used to influence practice, education, research, and policy?**

- Future research should incorporate technical support and digital training to improve participation in online interventions for older adults with frailty.
- A large‐scale randomised controlled trial is needed to further evaluate the effects of the programme amongst community‐dwelling older adults with frailty.

## 1 Introduction

Frailty is an age‐related clinical syndrome characterised by an increased vulnerable state to stressor events (Clegg et al. 2013). Older adults with frailty have increasing risks of adverse events, such as falls, disability, hospitalisation and mortality (Chu et al. 2021). The decline in independence accompanied by frailty reduces sense of control and competence (Archibald et al. 2020; Nair et al. 2022; Søvde et al. 2022).

Older adults with frailty have reported lower levels of subjective well‐being compared with those who are non‐frail (Crocker et al. 2019; Fujii et al. 2022). A likely reason is that older adults with frailty experience loss of control, decreased capacity for daily activities and reduced social engagement (Archibald et al. 2020; Bunt et al. 2023; Søvde et al. 2022). Subjective well‐being encompasses individuals' cognitive and affective evaluations of their lives, including positive emotions, the absence of negative emotions and overall life satisfaction (Diener 1984). Subjective well‐being has been considered an essential indicator of successful ageing (Qin et al. 2024). Lower subjective well‐being is associated with higher mortality rates and increased risks of chronic diseases (Zaninotto and Steptoe 2019; Zhu et al. 2023). Older adults with frailty can experience subjective well‐being when engaging in activities of daily living, maintaining independence and participating in social interactions depending on their available resources and abilities (Hemberg et al. 2020; Pan et al. 2019).

However, no interventions are specifically designed to improve subjective well‐being of older adults with frailty. A systematic review and meta‐analysis has synthesised evidence about the effects of non‐pharmacological interventions on the psychological outcomes of older adults with frailty (Tao et al. 2023). Previous studies have primarily focused on alleviating depressive symptoms, and none of the included studies has assessed subjective well‐being (Tao et al. 2023). Therefore, how subjective well‐being amongst older adults with frailty can be improved is unclear.

Psychoeducation has been demonstrated to considerably improve subjective well‐being in various populations, including individuals with schizophrenia (Shinozaki et al. 2020), breast cancer patients and their caregivers (Cipolletta et al. 2019), and community‐dwelling adults aged 25 to 75 (Heintzelman et al. 2020). A systematic review has identified that psychoeducation effectively reduces psychological distress and post‐traumatic stress disorder in older adults (Cremers et al. 2022). However, psychoeducation is primarily conducted in clinical settings or community centres (Gallego 2024; Maltby et al. 2020). Older adults with frailty tend to be homebound because of limited mobility, fear of falls or other adverse health changes, preventing them from outdoor activities (Søvde et al. 2022). Attending interventions in person has been found to be a key barrier to intervention participation amongst older adults with frailty (Angulo et al. 2020).

Home‐based telehealth interventions delivered via group videoconferencing are feasible and acceptable for individuals with physical or psychological problems (Banbury et al. 2018). This set‐up allows participation in a familiar environment, overcoming transportation barriers and fear of uncertainty (Banbury et al. 2018). However, current applications of this delivery model in older adults with frailty have focused predominantly on health status monitoring rather than serving as a platform for intervention implementation (Linn et al. 2021). Developing digital literacy could be challenging for some older adults because of limited exposure to digital technologies and age‐related sensory impairment (Vercruyssen et al. 2023), raising concerns about intervention feasibility and participant acceptance. To address these gaps, the present study aims to evaluate the feasibility and acceptability of a home‐based psychoeducation (HOPE) programme delivered via videoconferencing amongst older adults with frailty.

This study was underpinned by self‐determination theory, which proposes that individuals have three psychological needs, namely autonomy, competence and relatedness (Ryan and Deci 2017). Satisfaction of psychological needs is essential for subjective well‐being and optimal physical and psychosocial functioning of individuals (Ryan and Deci 2017). The theory implies that the decline in subjective well‐being amongst older adults with frailty can be explained by the unmet psychological needs for autonomy, competence and relatedness. This HOPE programme aims to empower older adults with frailty by helping them identify their strengths in meeting their psychological needs.

## 2 Methods

### 2.1 Aim and Objectives

This study aims to examine the feasibility and acceptability of a HOPE programme amongst older adults with frailty. The objectives are as follows:

1. To investigate the feasibility of eligibility criteria based on eligibility and recruitment rates,
2. to assess the feasibility of the programme in terms of attrition rate,
3. to evaluate the acceptability of the programme amongst participants in terms of attendance rate and satisfaction score,
4. to explore participants' experiences with HOPE,
5. to assess the feasibility and acceptability of the outcome measures in the target population in terms of missing data rate for each outcome measure and
6. to examine the preliminary effects of the programme on outcomes measures.

### 2.2 Study Design

This study used a single‐blinded, two‐arm parallel randomised controlled trial design. Participants were randomly assigned to either the intervention or control group on a 1:1 ratio. The study was conducted from November 2023 to April 2024. It was reported following the Consolidated Standards of Reporting Trials guidelines for pilot and feasibility trials (Eldridge et al. 2016).

### 2.3 Study Setting

Participants were recruited from four non‐government organisations which provide community services to older adults across different locations in Hong Kong.

### 2.4 Participants

Individuals were eligible for the study if they were (1) aged 65 years or older; (2) categorised as frail using the FRAIL scale (≥ 3 out of 5) (Abellan Van Kan et al. 2008); (3) mentally competent as screened by the Abbreviated Mental Test (AMT ≥ 6); (4) able to read Chinese; (5) living at home and (6) able to use smartphones (e.g., sending messages, watching videos). To ensure effective intervention delivery, we excluded individuals with visual, hearing or language barriers affecting their communication. To minimise confounding, we also excluded those with weekly exercise exceeding 150 min and undergoing psychiatric treatment, taking antidepressant medications or participating in other studies. In addition, to minimise risk of adverse events during interventions, we excluded individuals with a prognosis of 6 months or less or conditions contraindicating physical exercise, as suggested in ViviFrail programme which is a widely used physical exercise programme design for older adults with frailty (Izquierdo 2019). Examples were acute myocardial infarction or recent unstable angina, uncontrolled atrial or ventricular arrhythmias, dissecting aortic aneurysm, severe aortic stenosis, endocarditis/acute pericarditis, uncontrolled hypertension, acute thromboembolic events, acute or severe heart failure, acute or severe respiratory failure, uncontrolled orthostatic hypotension, acute decompensated diabetes or uncontrolled hypoglycaemia. Moreover, we excluded those with a fracture in the past month, have undergone major surgery in the past 6 months or are scheduled for major surgery in the next 6 months.

A minimum of 12 participants per group (intervention and control) is recommended for pilot studies when no prior information is available (Julious 2005). Considering that no evidence of psychoeducation exists for older adults with frailty in home‐based settings, a sample size of 24 participants in total, with 12 in each group, was required for this pilot study.

### 2.5 Randomisation, Allocation Concealment and Blinding

After eligibility screening, block randomisation with a block of six was used to allocate eligible participants to the intervention or control groups. The randomisation sequence was generated by computer and performed by an independent researcher who was not involved in participant recruitment and allocation. The results of group allocation were concealed in sealed opaque envelopes and submitted to a research assistant, who then contacted the participants and informed them of their group assignments after baseline assessments.

Owing to the nature of the intervention, blinding participants and interveners was not applicable in this study. To minimise the potential bias from the outcome assessment, the outcome assessors were blinded to the allocation results before the completion of the follow‐up outcome assessments. The participants were instructed to refrain from disclosing their group assignment to the outcome assessors. No instances of assessor unblinding were reported.

### 2.6 Intervention

#### 2.6.1 HOPE Programme

Participants in the intervention group engaged in weekly group‐based online psychoeducation sessions over 12 weeks. The dose of intervention was informed by the study of Heintzelman et al. (2020), which indicates that 12 weekly psychoeducation sessions could effectively improve subjective well‐being amongst community‐dwelling adults. The group‐based design was adopted because the findings of our systematic review suggest it may be a key intervention characteristic for reducing depressive symptoms in older adults with frailty (Tao et al. 2023). Each group consisted of six participants to ensure individual attention from the instructor (Sarkhel et al. 2020). Participants used smart devices such as tablets or smartphones to join sessions via Zoom, a widely used videoconferencing software, which has been proven user‐friendly for older adults (Shapira et al. 2021; Yeshua‐Katz et al. 2023). A tablet with a data SIM card was provided to participants who did not have internet access or devices.

The intervention content was designed based on the ‘Five Ways to Well‐Being’ framework. It is a set of five evidence‐based strategies, namely, ‘Connect’, ‘Be Active’, ‘Take Notice’, ‘Keep Learning’ and ‘Give’, for promoting subjective well‐being (Aked et al. 2008). Table 1 illustrates how the framework matches with the psychological needs for competence, autonomy and relatedness in self‐determination theory. ‘Be active’ and ‘Keep learning’ focused on building competence by promoting physical activity, home‐based exercise and lifelong learning. ‘Connect’ and ‘Give’ strengthened relatedness by encouraging social interactions, effective communication and acts of kindness. ‘Take notice’ was designed to foster autonomy by using mindfulness practice to enhance self‐awareness and self‐regulation, supporting more intentional choices in daily routines rather than acting automatically.

**TABLE 1:** Intervention outline.

Week	Topic^a^	Components of theoretical framework	Objectives	Content
1	Introduction	Competence	− Understand the concepts of health and healthy ageing − Learn warm‐up exercises	− Overview of the programme − Ice‐breaking exercises − Definition of health and healthy ageing − Strategies for healthy ageing − Instruction for warm‐up exercises *(Head, neck, ankle, trunk movements and back extension)*
2	Be active	Competence	− Recognise the importance of physical activities − Identify types of physical activities suitable for older adults − Develop a personal plan for increasing physical activity − Learn stretching exercises	− Overview of physical activity types and their benefits − Introduction to non‐exercise physical activities for daily life − Goal setting for ‘Stay active’ − Instruction stretching exercises *(Upper limbs stretching, lower limbs stretching)*
3	Be active	Competence	− Reflecting on the experience and barriers of physical activities − Identify the resource for overcoming barriers of physical activities	− Sharing experiences and challenges in physical activities − Introduction of resources for overcoming barriers to engaging in regular physical activities − Review for warm‐up and stretching exercises
4	Connect	Relatedness	− Recognise the importance of social connections − Identify methods for maintain social connections − Develop a personal plan for social connections − Learn breathing exercises	− Overview of social connections and their benefits − Sharing experience of seeking help − Introduction of social activities and for older adults − Goal setting for ‘Connect’ − Instructions for breathing exercises
5	Connect	Relatedness	− Reflecting on the experience and barriers of maintaining social connections − Identify the resource for overcoming barriers of social connections − Learn upper limb strength exercises	− Sharing experiences and challenges in maintaining social connections − Introduce community resources and communication skills for effective social connection − Instructions for upper limb strengthening exercises (*Biceps curl, Triceps curl, Lifting bottle(s) of water*)
6	Take notice	Autonomy	− Recognise benefits of mindfulness − Learn basic mindfulness practices − Develop a personal plan to mindfulness practices	− Overview of mindfulness and their benefits − Introduction of mindfulness practices (mindful breathing, body scan meditation) − Goal setting for ‘Take notice’ − Review for upper limb strengthening exercise
7	Take notice	Autonomy	− Reflecting the experience and challenges of mindfulness practice − Identify method for incorporating mindfulness to daily activities − Learn lower limb strengthening exercises	− Sharing experiences and challenges of mindfulness practices and the perceived effects − Introduction of mindfulness into daily life (mindful eating, gratitude journal) − Instructions for lower limb strengthening exercises *(Leg curl, Sit and stand with support, calf raises with support)*
8	Keep learning	Competence	− Recognise the importance of life‐long learning − Identify learning resource for older adults − Develop a personal learning plan	− Overview of lifelong learning and their benefits − Introduction of learning strategies suitable for older adults − Goal setting for ‘Keep learning’ − Review for lower limb strengthening exercise
9	Keep learning	Competence	− Reflecting the experience and challenges of learning − Introduce balance exercises	− Sharing experiences and challenges of keep learning in daily life − Introduction of resources for overcoming barriers in keep learning for older adults − Instructions for balance exercises *(Knee bends, sideways walking with support, tandem stance, one‐leg stand with support)*
10	Give	Relatedness	− Recognise the importance of giving − Identify practices of giving in daily life − Develop a personal plan for giving	− Overview of giving and its benefits − Introduction of practices of giving − Goal setting for ‘Give’ − Review for balance exercises
11	Give	Relatedness	− Reflecting the experience and challenges of giving in daily life − Identify method for incorporating giving to daily activities.	− Sharing experiences and challenges of giving in daily life − Discuss the method to incorporate giving to daily activities − Review for warm‐up exercise, stretching exercise, breathing exercises, strengthening exercises and balance exercises.
12	Closing		− Review and reflect on the ‘Five Ways to Well‐being’ approach − Establish personalised plans for continued well‐being practices and exercise	− Reflection of personalised well‐being and exercise plans

^a^ The topic is developed based on Five ways to Well‐being.

Each session lasted for 60 min. The first session provided an overview of the programme. Sessions 2 to 11 were based on the ‘Five Ways to Well‐Being’ framework, with each strategy covering over two sessions. The first session of each strategy began with an introduction of the strategy concerned in the ‘Five Ways to Well‐Being’ framework. This session was followed by discussion on how the strategy can be integrated into daily life. Participants identified their strengths through group discussions and then developed personalised weekly plans to apply them. In the following session, participants were encouraged to share their experiences and barriers in applying the strategies. The intervener then introduced different resources that would enable them to overcome the barriers. Specifically, the strategy of ‘Be Active’ spanned over all sessions to facilitate progressive learning of different types of home‐based physical activities. The last 20 min of all sessions were reserved for introducing or reviewing physical activities. All topics covered were reviewed in the final session.

#### 2.6.2 Attention Control

An attention control group was designed in this study to control potential confounding variables and reduce the attrition rate (LaFave et al. 2019). Participants in the control group received twelve 60‐min online group sessions from the intervener, matching the intervention group in dose, frequency, format and delivery mode to ensure equal contact time and attention (Aycock et al. 2018). However, the content of the sessions for the control group focused exclusively on information related to physical health because of ageing, covering topics such as healthy diet, gut health, incontinence, chronic pain management, fall prevention, oral health, sleep health, vision health, hearing health and home‐based exercise instructions.

### 2.7 Intervention Fidelity

The programme was developed by the first author, a registered nurse who has received certified training in positive psychology, mental health aid and psychoeducation. Two research assistants with a Bachelor of Social Science degree served as interveners. They were required to deliver the intervention according to a standardised protocol. The first author provided approximately 4 h of briefing to the two interveners, covering the intervention overview, session content and operational guidelines for using the Zoom platform. Furthermore, the first author monitored the interveners' adherence to the protocol in four randomly selected sessions (Breitenstein et al. 2010). If any confusion or divergence from the protocol arises, a discussion would be conducted between the first author and interveners immediately after sessions. The interveners provided each participant with a 20‐min face‐to‐face tutorial on using Zoom immediately after the baseline assessment at participants' homes. Participants were required to provide a return demonstration of how to use the tablet and Zoom during the tutorial. A leaflet with visual illustrations of Zoom functions was also provided.

### 2.8 Feasibility

The feasibility was defined by the eligibility rate, recruitment rate and attrition rate at 12 weeks post‐allocation follow‐up in this study. The eligibility rate was calculated as the proportion of participants meeting the inclusion criteria out of the total assessed. The recruitment rate was determined as the proportion of participants who consented out of the eligible individuals. The attrition rate was calculated as the percentage of participants lost to follow‐up at 12 weeks post‐allocation, based on the total initially assigned to the study. The first author contacted participants who dropped out from the study to understand the reasons for their attrition.

### 2.9 Acceptability

The acceptability was defined by the attendance rate to the intervention sessions and mean satisfaction scores of the intervention in this study. A 5‐item satisfaction survey was conducted by the end of the study for participants in the intervention group to rate the intervention from 1 (very dissatisfied) to 5 (very satisfied). The categories were appropriateness of the session content, length of each session, overall intervention duration and delivery mode.

Individual semi‐structured interviews were conducted with 11 participants in the intervention group to explore their experience of the HOPE programme. Interviews were conducted at the participants' homes by two trained research assistants. An interview guide was developed (Table 2). All interviews were audio‐taped for verbatim transcription.

**TABLE 2:** Interview guide.

Can you share what it was like to participate in this home‐based psychoeducation programme? What benefits or effects did you perceived since joining this programme? Why? What challenge or difficulty you faced whilst participating in this programme? How did you overcome those challenges or difficulties? In your opinion, how the programme can be improved?

### 2.10 Criteria for Proceeding to a Definitive Trial

The study could progress to a definitive trial if: (1) ≥ 20% of persons screened are eligible, (2) ≥ 60% of eligible participants are recruited, (3) attrition rate < 25% at 12 weeks, (4) missing data < 10% for each outcome measure, (5) attendance rate > 80% in the intervention group and (6) mean satisfaction score is over 3.5 out of 5 for all items.

### 2.11 Outcome Measures

Subjective well‐being and outcomes related to physical and psychosocial health were measured at baseline (T0) and 12 weeks post‐allocation (T1).

#### 2.11.1 Subjective Well‐Being

Subjective well‐being was measured by the 5‐item World Health Organization (WHO) Well‐being Index (World Health Organization 1998). It consists of five positive‐phrased items with a 6‐point Likert‐type scale ranging from 0 to 5. A higher score on the scale indicates a greater level of subjective well‐being, with a cut‐off score below 13 indicating low subjective well‐being. The Chinese version of WHO‐5 has good internal consistency, with Cronbach's *α* ranging from 0.81 to 0.86 (Fung et al. 2022).

#### 2.11.2 Physical Health

##### 2.11.2.1 Activities of Daily Living

Katz Index of Independence in Activities of Daily Living (ADL) (Katz et al. 1970) and Lawton Instrumental Activities of Daily Living Scale (IADL) (Graf 2008) were used to assess the independence of participants. The Katz Index of Independence in ADL is a widely used tool that assesses basic self‐care abilities, including bathing, dressing, toileting, transferring, continence and feeding. The Lawton IADL Scale focuses on assessing an individual's ability to perform complex activities that are necessary for independent living, such as handling finances, using transportation, preparing meals, handling medications, housekeeping, doing laundry, shopping and using the telephone. Higher scores in ADL and IADL indicate greater independence in performing self‐care activities and instrumental activities, respectively. Both scales have been validated amongst Chinese community‐dwelling older adults (Tang et al. 1999).

##### 2.11.2.2 Physical Fitness

Physical fitness was assessed using the Senior Fitness Test (SFT) battery, developed and validated by Rikli and Jones (2013). The SFT includes seven tests: the 30‐s chair stand (lower body strength), 30‐s arm curl (upper body strength), back scratch (upper body flexibility), chair sit‐and‐reach (lower body flexibility), 8‐ft up‐and‐go (agility), 2‐min step‐in‐place (aerobic capacity) and body mass index. SFT is a reliable and validated tool for assessing physical functioning amongst older adults in the community (Xu et al. 2022).

#### 2.11.3 Psychosocial Health

##### 2.11.3.1 Depressive Symptoms

The 4‐item Geriatric Depression Scale (GDS‐4) was used to assess participants’ depressive symptoms, with a score range of 0 to 4 (Yesavage and Sheikh 1986). The GDS‐4 includes four yes or no questions, with higher scores indicating a greater presence of depressive symptoms. The GDS‐4 has been validated amongst Chinese community‐dwelling older adults (Cheng and Chan 2008).

##### 2.11.3.2 Social Support

The social support was measured by the 10‐item Duke Social Support Index (Landerman et al. 1989). Scores range from 0 to 30, with higher scores indicate better perceived social support. The DSSI‐10 has good internal consistency amongst Chinese older adults (Cronbach's *α* = 0.77) (Li et al. 2015).

##### 2.11.3.3 Health‐Related Quality of Life

Health‐related quality of life was assessed using the EuroQol 5‐Dimension 5‐Level (EQ‐5D‐5L) questionnaire, developed and validated by the EuroQol Group (1990). The EQ‐5D‐5L includes five dimensions: mobility, self‐care, usual activities, pain or discomfort and anxiety or depression, each with five levels of severity. The EQ‐5D‐5L has shown good validity and reliability amongst Chinese older adults (You et al. 2020).

#### 2.11.4 Socio‐Demographic Data

Socio‐demographic data, including age, sex, marital status, education level, living status, number of medications and comorbidity were collected. The presence of comorbidity was recorded using the Charlson Comorbidity Index (Charlson et al. 2022).

### 2.12 Data Collection

Staff members of the participating organisations introduced the study to their clients. Interested individuals then completed eligibility screening through an online survey. The first author approached those who were eligible and explained the study's purpose, procedures and requirements through phone. A face‐to‐face meeting was scheduled with eligible individuals to obtain written consent and conduct a baseline assessment at the participants' homes. A follow‐up assessment was conducted at the participants' homes at 12 weeks by two trained research assistants who were blinded to group assignment of participants. Allocation was disclosed to the assessor only after completion of all follow‐up assessments for each participant to determine eligibility for a post‐intervention semi‐structured interview.

### 2.13 Data Analysis

Descriptive statistics were used to summarise the demographic characteristics and study outcomes of the participants. The intention‐to‐treat principle was used in the analysis. Normality was assessed by visual inspection of Q–Q plots, indicating non‐normality distribution of all outcomes. Therefore, the Mann–Whitney U test and Wilcoxon signed‐rank test were used to compare the group differences and differences in study outcomes across time, respectively. Bootstrapped linear regressions adjusting for baseline were conducted to estimate group difference in outcomes under non‐normality. Statistical analysis was conducted using IBM SPSS v26.0. All statistical tests were two‐tailed with a 0.05 level of statistical significance. The interview transcripts were analysed using qualitative content analysis (Graneheim and Lundman 2004). The authors read and coded the transcripts independently. Related codes were grouped into broader categories to increase understanding of participants' experiences of intervention. When disagreements arose during the analysis, the authors reflected on different interpretations of data and discussed to reach a consensus.

### 2.14 Ethics Consideration

This study has been approved by the New Territories East Cluster Clinical Research Ethics Committee (Approval no: 2023–585). This study was conducted in compliance with the International Conference on Harmonisation of Good Clinical Practice (ICH‐GCP) and the Declaration of Helsinki. Assessments and interventions were not performed before obtaining written informed consent from the eligible participants. All participation was voluntary, and participants had the right to withdraw from the study at any time. To maintain confidentiality, unique numbers were used to identify participants in this study. All physical data were stored in a locked cabinet and only accessible to the researchers in this study. All data will be destroyed 3 years after completion of the study.

## 3 Results

### 3.1 Subject Recruitment

**FIGURE 1:**
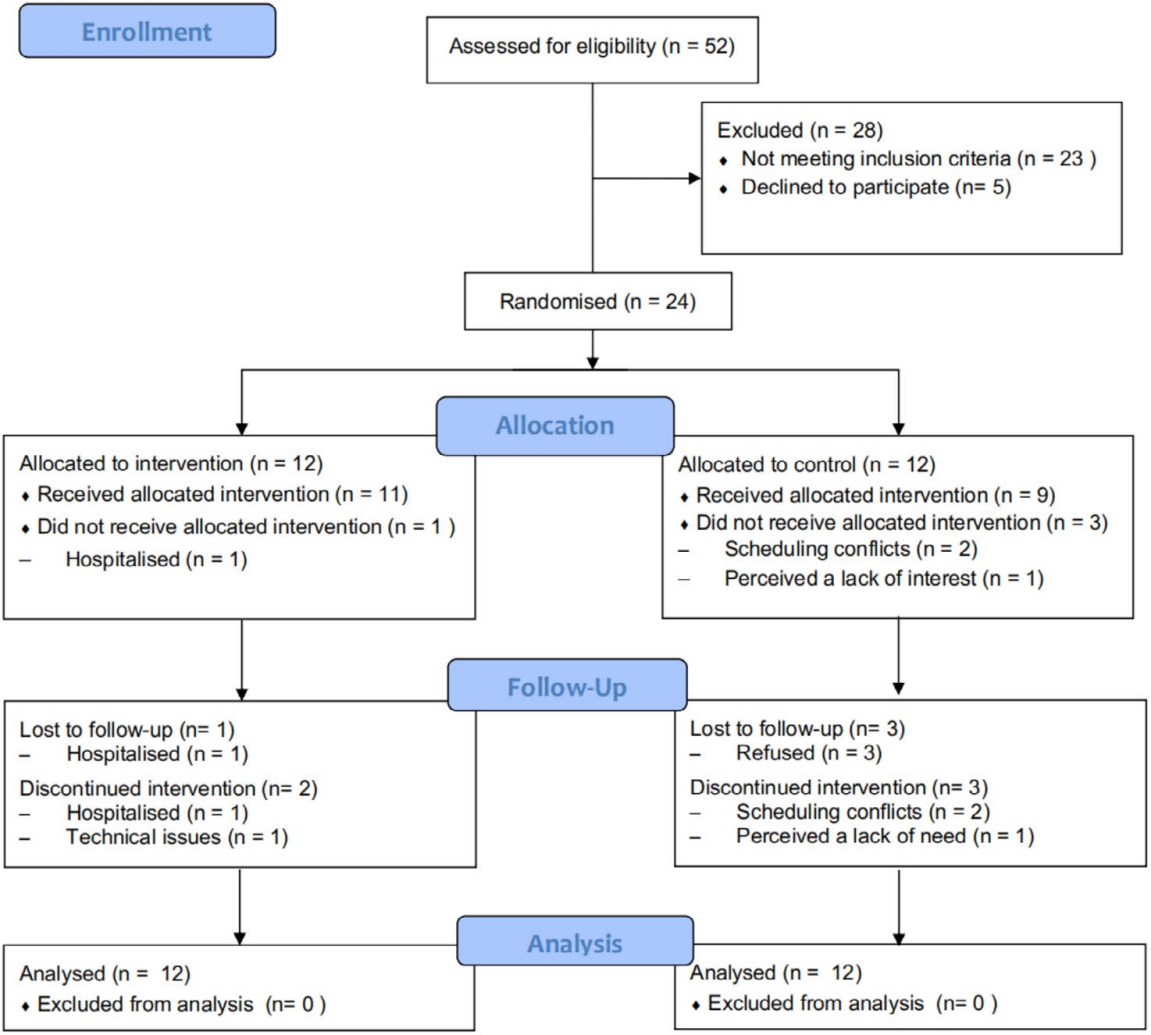
CONSORT flow diagram of the study.

Fifty‐two older adults were approached for eligibility screening. Twenty‐nine individuals met the eligibility criteria, with an eligibility rate of 55.8%. Of these, five individuals declined to participate, giving a recruitment rate of 82.8%. The reasons for refusal included fatigue (*n* = 2), lack of interest (*n* = 2) and scheduling conflicts (*n* = 1). A final total of 24 participants were recruited in this study (Figure 1).

### 3.2 Characteristics of Participants

The mean age of participants was 77.7 ± 6.2, ranging from 66 to 89 years old, and most participants were female (91.7%). More than half of them were widowed (54.2%), had an education level of middle school or above (62.5%) and lived with family or domestic worker (54.2%). The mean number of medications taken by the participants was 3.1 ± 1.8, and the mean of the Charlson Comorbidity Index score was 4.5 ± 2.0 (Table 3). All participants from the intervention group were interviewed, except one who withdrew from the study because of hospitalisation. The mean age of interviewees was 77.9 ± 5.7, with a range of 71 to 87 years. The majority were female (81.8%), over half were widowed (54.5%) and lived alone (54.5%). Nearly half had attained a primary level of education (42.9%). Their changes in the 5‐item WHO Well‐being Index scores over the 12‐week follow‐up period ranged from −1 to 15 compared to baseline (Table 4).

**TABLE 3:** Participants' characteristics.

	All (*N* = 24)	Intervention group (*n* = 12)	Control group (*n* = 12)
	*n* (%)	*n* (%)	*n* (%)
Age (years), mean (SD)	77.7 (6.2) [range: 66–89]	78.8 (6.3)	76.6 (6.2)
Sex			
Male	3 (12.5)	2 (20.2)	1 (26.3)
Female	21 (91.7)	10 (79.7)	11 (73.7)
Marital status			
Married	7 (29.2)	3 (25.0)	4 (33.3)
Widowed	13 (54.2)	7 (58.3)	6 (50.0)
Single/Divorced	4 (16.7)	2 (16.7)	2 (16.7)
Education level			
Primary school	9 (37.5)	5 (41.7)	4 (33.3)
Middle school	6 (25.0)	2 (16.7)	4 (33.3)
High school	6 (25.0)	4 (33.3)	2 (16.7)
Tertiary level	3 (12.5)	1 (8.3)	2 (16.7)
Living status			
Living alone	11 (45.8)	7 (58.3)	4 (33.3)
Only with partner	5 (20.8)	1 (8.3)	4 (33.3)
Only with children	4 (16.7)	1 (8.3)	3 (25.0)
With children and partner	1 (4.7)	1 (8.3)	0
With domestic worker	3 (12.5)	2 (16.7)	1 (8.3)
Number of medication, mean (SD)	3.1 (1.8)	3.1 (1.7)	3.2 (2.0)
Total CCI score, mean (SD)	4.5 (2.0)	4.6 (2.0)	4.3 (2.1)

*Note:* Presented in number (percentage), unless specified.

Abbreviation: CCI, Charlson Comorbidity Index.

**TABLE 4:** Participants' characteristics (*n* = 11).

Code	Sex	Age	Marital status	Educational level	Living status	Changes in WHO‐5 between T0 and T1
001	Female	76	Widowed	Secondary	Alone	5
002	Female	77	Widowed	Primary	Alone	2
008	Female	82	Married	Secondary	With children and partner	13
011	Female	75	Widowed	Primary	Alone	5
014	Female	86	Widowed	Primary	Alone	4
016	Female	71	Divorced	Secondary	Alone	1
019	Female	87	Widowed	Tertiary	With domestic	15
020	Male	72	Married	Primary	Alone	10
023	Male	76	Married	Secondary	With partner	−1
024	Female	83	Widowed	Primary	With children	−1
025	Female	72	Single	Secondary	With domestic	9

Abbreviations: T0, baseline; T1, 12‐week post‐allocation; WHO‐5, 5‐item WHO Well‐being index.

### 3.3 Feasibility of the Study

The attrition rate of this study was 16.7%. One participant from the intervention group quit the study because of hospitalisation. Three participants from the control group withdrew as a result of scheduling conflicts (*n* = 2) and perceived a lack of interest for the study (*n* = 1) (Figure 1).

### 3.4 Acceptability of the Study

Eight participants in the intervention group completed all sessions, with a mean attendance rate of 81%. Two participants missed two sessions because of schedule conflicts, and two discontinued with attendance rates below 80% due to technical issues and hospitalisation (Figure 1). Eleven participants assigned to the intervention group completed the satisfaction survey and reported a mean satisfaction score of items ranging from 4.4 to 4.6 out of 5. No adverse events were reported. No missing data were reported in all outcomes at baseline and 12 weeks follow‐up. All prespecified progression criteria for proceeding to a definitive trial were met (Table 5).

**TABLE 5:** Assessment of progression criteria for a definitive trial.

Criterion for proceeding to a definitive trial	A priori threshold	Result	Met?	Notes
(1) Eligibility rate	≥ 20%	55.8%	Yes	Eligible/screened
(2) Recruitment rate	≥ 60%	82.8%	Yes	Recruited/eligible
(3) Attrition rate at 12 weeks	< 25%	16.7%	Yes	Attrition at 12 weeks
(4) Missing data for each outcome measure amongst completers	< 10% (each outcome)	0%	Yes	Calculated amongst participants who completed baseline assessment and 12‐week follow‐up; attrition is reported separately in (3)
(5) Attendance rate in the intervention group	> 80%	81.0%	Yes	Attendance rate (intervention group)
(6) Mean satisfaction score (all items)	> 3.5/5 (all items)	4.4–4.6	Yes	Range across items; all items > 3.5

### 3.5 Potential Effects of the Intervention

Median WHO‐5 scores were 13.0 at T0 and 10.0 at T1 in the control group, and 10.0 at T0 and 17.0 at T1 in the intervention group. Participants in the intervention group had significant improvements in subjective well‐being (*Z* = −2.585, *p* = 0.010) and agility (*Z* = −2.497, *p* = 0.013) at 12 weeks post‐allocation. No significant improvement in outcomes was found in the control group (Table 6). No significant group difference was observed in outcomes at 12 weeks post‐allocation (Table 7). After adjusting for baseline, the intervention group showed significantly higher WHO‐5 scores than the control group at T1 (*B* = 4.29, SE = 1.79, 95% CI [0.91, 7.76], *p* = 0.036) (Table 8).

**TABLE 6:** Pre‐ and post‐intervention analysis using Wilcoxon Signed Rank test.

	Intervention group (*n* = 11)		Control group (*n* = 10)	
	*Z*	*p*	*Z*	*p*
WHO‐5	−2.585	0.010	−0.598	0.550
GDS‐4	−0.137	0.891	−0.175	0.861
DSSI‐10	−1.491	0.136	−1.131	0.258
EQ‐5D‐5L	−0.978	0.328	−0.224	0.823
Katz ADL	−1.121	0.262	−0.577	0.564
Lawton IADL	−1.625	0.104	−0.816	0.414
Senior fitness test				
30 s Arm curl test (no. of reps)	−1.192	0.233	−0.537	0.591
30 s Chair stand test (no. of stands)	−0.516	0.606	−0.493	0.622
Back scratch test (cm)	−0.356	0.722	−1.051	0.293
Chair sit and reach test (cm)	−0.892	0.373	−0.314	0.753
Agility: 8‐ft up and go test (s)	−2.497	0.013	−1.481	0.139
2‐min step test (no. of steps)	−1.468	0.142	−1.245	0.213
BMI	−1.334	0.182	−0.059	0.953

Abbreviations: ADL, activities of daily living; BMI, body mass index; DSSI‐10, 10‐item Duke Social Support Index; EQ‐5D‐5L, EuroQol 5‐Dimension 5‐Level; GDS‐4, 4‐item Geriatric Depression Scale; IADL, instrumental activities of daily living; no. of reps, number of repetitions; WHO‐5, 5‐item WHO Well‐being index.

**TABLE 7:** Comparison of outcomes between groups at baseline and 12 weeks post‐allocation.

	Intervention group	Control group	*p*
	Mean (SD)	Mean (SD)	
WHO‐5			
T0	11.8 (6.0)	13.7 (5.5)	
T1	16.6 (4.0)	13.9 (6.9)	0.313
GDS‐4			
T0	0.9 (1.3)	1.3 (1.3)	
T1	0.9 (0.8)	1.4 (1.5)	0.443
DSSI‐10			
T0	23.6 (2.5)	23.3 (4.6)	
T1	24.5 (3.4)	21.3 (5.9)	0.182
EQ‐5D‐5L			
T0	0.6 (0.3)	0.6 (0.3)	
T1	0.6 (0.2)	0.7 (0.3)	0.247
Katz ADL			
T0	10.8 (1.9)	11.8 (0.4)	
T1	11.4 (0.9)	11.9 (0.3)	0.104
Lawton IADL			
T0	6.0 (2.0)	7.3 (1.1)	
T1	7.0 (1.3)	7.3 (1.0)	0.529
Senior fitness test			
30 s Arm curl test (no. of reps)			
T0	9.4 (4.5)	10.9 (5.2)	
T1	11.0 (3.4)	10.1 (5.4)	0.660
30 s Chair stand test (no. of stands)			
T0	8.4 (2.9)	9.6 (4.7)	
T1	8.4 (2.9)	11.2 (5.8)	0.218
Back scratch test (cm)			
T0	−11.9 (12.2)	−12.3 (14.1)	
T1	−7.9 (9.6)	−6.2 (9.3)	0.690
Chair sit and reach test (cm)			
T0	−10.4 (9.5)	−6.8 (10.2)	
T1	−8.3 (9.0)	−3.5 (5.5)	0.166
Agility: 8‐ft up and go test (s)			
T0	16.8 (12.0)	12.6 (7.6)	
T1	12.7 (6.4)	10.7 (5.2)	0.491
2‐min step test (no. of steps)			
T0	58.9 (19.7)	77.4 (29.4)	
T1	67.4 (18.8)	88.5 (28.0)	0.059
BMI			
T0	24.8 (5.4)	24.5 (3.8)	
T1	24.0 (4.9)	23.5 (4.0)	0.816

Abbreviations: ADL, activities of daily living; BMI, body mass index; DSSI‐10, 10‐item Duke Social Support Index; EQ‐5D‐5L, EuroQol 5‐Dimension 5‐Level; GDS‐4, 4‐item Geriatric Depression Scale; IADL, instrumental activities of daily living; no. of reps, number of repetitions; WHO‐5, 5‐item WHO Well‐being index.

**TABLE 8:** Bootstrap regression results of group differences in outcomes at 12 weeks post‐allocation adjusted for baseline.

Outcomes	(*B*)	Bootstrap (SE)	95% CI	*p*
WHO‐5	4.29	1.79	[0.91, 7.76]	0.036
GDS‐4	−0.40	0.61	[−1.61, 0.85]	0.520
DSSI‐10	2.33	1.29	[−0.36, 5.02]	0.093
EQ‐5D‐5L	−0.17	0.11	[−0.37, 0.08]	0.162
Katz ADL	−0.23	0.23	[−0.60, 0.16]	0.371
Lawton IADL	0.07	0.37	[−0.64, 0.73]	0.866
Senior fitness tests				
30 s Arm curl test (no. of reps)	0.96	1.47	[−1.54, 3.58]	0.521
30 s Chair stand test (no. of stands)	−2.14	1.92	[−6.82, 1.95]	0.324
Back scratch test (cm)	−2.62	3.69	[−10.44, 6.07]	0.521
Chair sit and reach test (cm)	−2.92	3.02	[−9.34, 3.68]	0.398
Agility: 8‐ft up and go test (s)	0.37	1.52	[−2.31, 2.96]	0.832
2‐min step test (no. of steps)	−12.98	10.69	[−34.52, 13.83]	0.276
BMI	0.36	0.46	[−0.57, 1.22]	0.450

Abbreviations: ADL, activities of daily living; BMI, body mass index; DSSI‐10, 10‐item Duke Social Support Index; EQ‐5D‐5L, EuroQol 5‐Dimension 5‐Level; GDS‐4, 4‐item Geriatric Depression Scale; IADL, instrumental activities of daily living; no. of reps, number of repetitions; WHO‐5, 5‐item WHO Well‐being index.

### 3.6 Qualitative Findings

The qualitative interview results identified three themes, namely, facilitators of intervention implementation, perceived benefits and challenges of home‐based psychoeducation (Table 9).

**TABLE 9:** Qualitative findings.

Themes	Subthemes
Facilitators of intervention implementation	Convenience
	Clear visual illustration
Perceived benefits	Gaining a sense of accomplishment
	Being connected
	Feeling empowered
Challenges of HOPE	Low digital literacy
	Schedule conflicts
	Comprehension barriers
	Retention difficulties

#### 3.6.1 Facilitators of Intervention Implementation

Participants' sharing revealed the facilitators of home‐based psychoeducation implementation.

##### 3.6.1.1 Convenience

Many participants highlighted the convenience of the online home‐based delivery mode because it reduced barriers associated with limited mobility. For example, one participant said,

The online format is extremely beneficial for people like us with limited mobility. It's good because [we] don't need to go out. My son also worries about [me] when [I] go out sometimes. (019)

##### 3.6.1.2 Clear Visual Illustration

Some participants appreciated the illustration for improving their understanding and retention of session content in the intervention booklet. These visual illustrations were particularly helpful in clarifying concepts not fully understood during sessions. For example, one participant said that,

First, when I received your booklet, ‘The Five Ways to Well‐being,’ I thought it was excellently crafted. It features illustrations and text that are clear and easy to understand, allowing us to grasp the content immediately…We might not grasp everything all at once, [but] there are written explanations, exercise demonstrations (video), and pictures…it's easy to accept, and I really like it. (019)

#### 3.6.2 Perceived Benefits

Participants appreciated positive changes in their lives after the intervention. These changes improved their level of engagement in daily life and ability to rebound from life challenges.

##### 3.6.2.1 Gaining a Sense of Accomplishment

Participants reported a sense of accomplishment from their improved physical functioning. Previously, they avoided daily activities because of negative perceptions about their physical functioning. However, the programme helped them recognise their ability to engage in physical activities based on their strengths. This improvement motivated them to participate more actively, further enhancing their sense of achievement. For example, one participant reported,

I feel much lighter overall now. I couldn't even walk before, but now I can make it up to the second floor and climb the stairs. I'm quite satisfied with [it]. (025)

##### 3.6.2.2 Being Connected

Several participants expressed their enjoyment of interactions with others during the intervention. Communicating with peers in similar situations provided a sense of familiarity and enhanced their understanding of their own circumstances. Intergenerational interactions between participants and the instructor during sessions also made participants feel connected and supported by society. For example, one participant explained,

Many people were willing to speak up [their stories] in our discussion, which was very good… In these Zoom sessions, everyone was willing to talk. Sometimes, I might not say [it], but I found that I was much better than many others. I realised that some of them are so isolated or have such limited activities. (016)

Another participant added,

The feeling of being with young people is different, and it makes me very happy… Having you [guys] around and seeing your youthful energy makes us happy, so I'm truly grateful… I feel happier and in a better mood, and I feel I'm not forgotten by society. (019)

##### 3.6.2.3 Feeling Empowered

Some participants mentioned that the programme broadened their perspectives on daily life, helping them identify more options for activities and adopt new ways of thinking. They became more proactive in engaging in their daily activities. One participant shared that,

The ‘Five Ways to Well‐being’ reminded us of many things we had overlooked and provided different ideas for learning and activities. For example, ‘Be Active’, you taught us that we could do something like gardening, which we would never have thought before. When we immerse ourselves in these activities, our mood changes, and we are able to shift our mindset through them. (016)

#### 3.6.3 Challenges of HOPE

Participants highlighted challenges they encountered whilst attending the intervention. These challenges are primarily associated with technical issues, a fixed session schedule and comprehension and retention of psychoeducation content.

##### 3.6.3.1 Low Digital Literacy

Some participants experienced unexpected technical issues during the sessions, such as being disconnected from the Zoom meeting, and were uncertain about how to resolve them. This lack of technical skills hindered their participation. One participant who discontinued intervention because of technical issues told that,

Even though I press [the button], I still do not know what's happening. [Sometimes] it just didn't work… you've told me before to press there, and after pressing, it can enter the session by itself. But sometimes, no matter how I press, I can't get into [the sessions], and I had no idea what was wrong. (014)

##### 3.6.3.2 Schedule Conflicts

Several participants highlighted the effects of scheduling conflicts on their attendance of sessions, particularly because of frequent medical appointments related to chronic illnesses. The lack of flexibility in the group‐based session made it difficult for them to participate consistently. One participant who missed two sessions mentioned that

I spent a lot of time going to the doctor… Sometimes, there were conflicts between [appointment] and the session schedule, especially when I booked them and forgot… It's just that sometimes schedules need to be adjusted. (002)

##### 3.6.3.3 Comprehension Barriers

Several participants reported difficulties in grasping the abstract content of the psychoeducation, especially for the topic of ‘Take notice’. They suggested that more concrete examples would aid in better understanding and application of the session content. For instance, one participant noted,

Sometimes, it was hard for me when you asked me to give examples (of mindfulness). I didn't know what to say… Even when you asked us what strategies we have adopted, I didn't know how to respond immediately… I found it a bit complicated. (008)

##### 3.6.3.4 Retention Difficulties

Participants mentioned that they quickly forgot the content after learning it. One participant said that

I couldn't remember things. I forget what I learn almost immediately. I have a poor memory, and things just did not get into my brain… It's just that I don't remember it afterwards. (011)

## 4 Discussion

This study is the first to examine the feasibility and acceptability of a HOPE programme for community‐dwelling older adults with frailty. The programme shows good feasibility and acceptability in terms of delivery mode, dose and session content, with good potential to enhance subjective well‐being in this population. Despite these positive findings, several challenges of implementing the programme were identified, including low digital literacy, schedule conflicts and difficulties in comprehension and retention of psychoeducation content.

This study indicates that the psychoeducation guided by self‐determination theory has potential in addressing psychological needs amongst older adults with frailty (Ryan and Deci 2017). Participants reported that the programme broadened their perspectives and helped them identify feasible activity options based on their abilities, facilitating more proactive engagement in daily activities. It suggest a greater sense of control over daily activity choices and participation amongst older adults with frailty. The strength‐based approach, which focuses on individuals' personal, social and community resources rather than deficits (Caiels et al. 2021), is crucial for older adults to maintain quality of life despite frailty (Dury et al. 2018). The sense of accomplishment from improved physical functioning and ability to perform previously challenging activities fulfilled participants' need for competence. Engaging in discussions and sharing experience with peers in similar situation allowed participants to learn from others' perspectives. Our qualitative findings also suggest that participation in the virtual group sessions involved social comparison processes. Participants reported feeling much better than other peers after hearing how isolated others were. It reflected downward comparison that can provide reassurance and a sense of coping. This aligns with the previous study (Locock and Brown 2010), which indicated that social comparison amongst peers can be both beneficial (e.g., reassurance, gratitude, hope) and harmful (e.g., distress, guilt or heightened awareness of vulnerability), depending on what is observed. In our study, this suggests that online group interactions may simultaneously provide connection and motivation whilst shaping participants' self‐evaluations through social comparison. Furthermore, our findings showed the intergenerational interactions between the interveners. The participants also contributed to a sense of connection amongst themselves. Previous reviews indicated interventions involving intergenerational interactions have positive benefits on older adults' outcomes, such as reduced depressive symptoms and increased subjective well‐being (Krzeczkowska et al. 2021; Zhong et al. 2020). The increased social interactions fostered a sense of connection and support, addressing their need for relatedness. These perceived benefits may explain the improvement in subjective well‐being observed amongst participants in the intervention group because the satisfaction of these needs is essential for optimal functioning and subjective well‐being (Ryan and Deci 2017).

Although previous research has demonstrated the good feasibility and effectiveness of the ‘Five ways to well‐being’ framework in the general population (Prydz et al. 2024), our study reveals that older adults with frailty still faced challenges in understanding and practicing the ‘Take notice’ aspect. This finding may be attributable to approximately 40% of participants having primary school education, potentially limiting their capacity to process abstract conceptual content. Therefore, providing participants with more targeted and practical approaches to better understanding and practicing these strategies are crucial. As suggested by our qualitative findings, visual demonstration could also be useful for supporting participants' understanding and retention of psychoeducation content because individuals achieve better learning outcomes when exposed to a combination of words and visual illustration, as opposed to words alone (Mayer 2002).

This study reveals that the psychoeducation delivered through videoconferencing mode is feasible and acceptable amongst community‐dwelling older adults with frailty. The attendance rate of the programme is consistent with a previous systematic review, which has indicated that attendance rate of home‐ group‐based group interventions via videoconferencing platform amongst other population ranges from 66% to 93.8% (Banbury et al. 2018). The qualitative findings indicate that the good acceptability may be attributed to convenience of a home‐based delivery mode via videoconferencing platform. Information and communication technologies such as videoconferencing have been increasingly used for frailty management, but most of them focused on physical function assessment, remote monitoring and physical exercise training (Dawson et al. 2023; Gallucci et al. 2021; Linn et al. 2021). Our study further reveals the potential of videoconferencing in offering remote psychological support to homebound older adults, such as those with severe mobility impairments or chronic conditions that restrict their outdoor activities. This delivery mode may also overcome constraints for in‐person services in the situation of pandemic, similar to the social distancing measures during COVID‐19 (Gallistl et al. 2021), or other public health emergencies. However, the low digital literacy of some participants also negatively affects their experience of HOPE. Negative feelings caused by technical problems could prevent older adults from actively engaging in digital technologies (Airola et al. 2020). Therefore, technical support for older adults should not be a one‐time arrangement. Instead, continuous technical support throughout the entire process of remote service is essential to ensure sustained participation and engagement.

### 4.1 Implications

This pilot study provides insights into practice and future research on HOPE for community‐dwelling older adults with frailty.

Several strategies are suggested to facilitate the implementation of HOPE programme amongst community‐dwelling older adults with frailty. Firstly, providing a hotline for participants is recommended to address unforeseen technical issues during session. Tutorial sessions can also incorporate training on common technical issues to equip participants with abilities to solve sudden technical issues. Secondly, the fixed schedule of group sessions can limit autonomy amongst older adults with frailty, who often require frequent healthcare services, including outpatient visits, emergency care and hospitalisations (Ge et al. 2020). Participants' healthcare schedules should be discussed with them before finalising the group‐based session schedule. To facilitate participants' comprehension of the topic, the practice should be broken down into small steps guiding participants through step‐by‐step instructions and reminders. Reviews of the previous session's content at the beginning of each session are recommended to support content retention.

This pilot study also reveals the feasibility and acceptability of the HOPE programme amongst older adults with frailty. The relatively low attrition rate, high attendance rate and satisfaction scores indicate that the programme have good feasibility and acceptability in terms of delivery mode, dose and session content. A definitive randomised controlled trial could be conducted to examine the effects of the HOPE on subjective well‐being amongst this population.

### 4.2 Limitations

We acknowledge several limitations of this study. All participants were recruited from NGO members who were more proactive and outgoing. It may overlook those who are homebound and socially isolated. Future research can consider recruiting participants from diverse sources, such as outpatient clinics, to increase the representativeness and generalisability of the findings. To ensure safety and feasibility of the intervention, this study excluded individuals with communication barriers, those receiving psychiatric treatment or taking antidepressants, and those with conditions contraindicating exercise. These exclusions may also limit the generalisability of our findings to frail older adults with sensory disability, mental health comorbidities and greater medical complexity. Future studies should explore adapted delivery formats and content and evaluate safety and effectiveness in more clinically heterogeneous samples. Although we implemented assessor blinding for outcome assessments, complete blinding across all study procedures was not feasible because qualitative interviews were conducted with intervention participants after follow‐up assessments. It might introduce social desirability and recall bias for the results. Besides, the study primarily involved female participants, which may lead to gender bias. Lastly, the intervention's potential effects should be treated with caution because of the small sample size.

## 5 Conclusion

The study indicates that a HOPE programme is feasible and acceptable for community‐dwelling older adults with frailty. This finding necessitates a definitive randomised controlled trial to evaluate its effect on subjective well‐being. Participants faced challenges such as lack of digital literacy, conflicts in scheduling and difficulties with comprehension and retention of content. To enhance implementation, the definitive study should provide a hotline for technical support, flexible scheduling, more practical examples of session content and a session review.

## Author Contributions

**An Tao:** conceptualization, data collection and analysis, writing original draft, and writing – review and editing. **Helen Yue Lai Chan:** conceptualization, data analysis, writing – review and editing, and supervision. **Sarah Xiao:** writing – review and editing. **Ken Hok Man Ho:** supervision.

## Ethics Statement

This study has been approved by the Joint Chinese University of Hong Kong–New Territories East Cluster Clinical Research Ethics Committee (Approval no: 2023–585).

## Conflicts of Interest

The authors declare no conflicts of interest.

## Data Availability

The data that support the findings of this study are available on request from the corresponding author. The data are not publicly available due to privacy or ethical restrictions.
